# The Impact of Differential Posterior Tibial Slope on Clinical Outcomes Following Revision ACL Surgery: A Retrospective Single-Centre Study

**DOI:** 10.3390/jcm15187125

**Published:** 2026-09-14

**Authors:** Konstantinos Tsikopoulos, Paul White, James Robinson, Konstantinos Kazamias, Luke Duggleby, Kamrul Hasan, Nick Howells, James Murray

**Affiliations:** 1 2nd Orthopaedic Department, National and Kapodistrian University of Athens, 11527 Athens, Greece; 2 Department of Mathematics and Statistics, University of the West of England, Bristol BS16 1QY, UK; paul.white@uwe.ac.uk; 3 Spire Hospital Bristol, Bristol BS6 6UT, UK; 4 Royal Orthopaedic Hospital NHS Foundation Trust, Birmingham B31 2AP, UK; k.kazamias@nhs.net; 5 North Bristol NHS Trust, Bristol BS10 5NB, UK; luke.duggleby@nbt.nhs.uk (L.D.); hasankamrul@hotmail.com (K.H.); nick.howells@nbt.nhs.uk (N.H.); james.murray@doctors.org.uk (J.M.)

**Keywords:** posterior tibial slope, interobserver variability, intraobserver variability, revision anterior cruciate ligament reconstruction

## Abstract

**Background:** Recent evidence has suggested that patients undergoing anterior cruciate ligament (ACL) revision surgery tend to have a larger differential posterior tibial slope compared to healthy individuals. The impact of differential slope on patient reported clinical outcomes following revision ACL surgery remains unclear. **Methods:** Patients undergoing revision ACL surgery between 2019 and 2024 in our institution were eligible for inclusion. The primary outcome was the change in subscales of the Knee injury and Osteoarthritis Outcome Score (KOOS) from preoperative to at least 1 year postoperative. Medial and lateral posterior tibial slopes were measured independently by four assessors on preoperative magnetic resonance imaging (MRI) scans. The correlation between change in KOOS and the mean differential posterior tibial slope (dPTS) was calculated by using Pearson’s correlation statistic. **Results:** Data from 31 revision ACL cases were analysed. There were statistically significant postoperative improvements in all five KOOS domains (Symptoms *p* = 0.036, Pain *p* = 0.004, Daily Living *p* = 0.002, Sport and Recreation *p* < 0.001, Quality of Life *p* < 0.001). There was a statistically significant correlation between mean differential slope and change in KOOS Symptoms (*p* = 0.007), Pain (*p* < 0.001), Sport and Recreation (*p* = 0.034), and Quality of Life (*p* = 0.010), but not with Daily Living (*p* = 0.070). **Conclusions:** In patients undergoing ACL revision reconstruction, greater differential posterior tibial slope is associated with poorer patient-reported outcomes. MRI-based assessment of dPTS demonstrated moderate single-rater and good average-rater interobserver reliability, potentially aiding in preoperative risk stratification and patient management.

## 1. Introduction

Graft rupture remains a serious adverse outcome after anterior cruciate ligament (ACL) reconstruction, leading to significant patient morbidity and substantial cost implications for healthcare systems. Several anatomical risk factors have been linked to an increased risk of ACL graft failure, including high body mass index (BMI), female sex, small graft diameter (<8 mm), malalignment, meniscal deficiency, and increased posterior tibial slope (PTS) [1,2]. Of these factors, PTS has received increasing attention due to its influence on increasing anterior tibial translation and ACL graft load [3]. Previous studies have shown that a lateral PTS ≥ 10° or medial PTS ≥ 14° is associated with a significantly higher risk of ACL graft failure and revision surgery [4,5]. Furthermore, long-term data indicate that increased PTS is not only associated with reduced ACL graft survivorship [6], but also increased risk of third-time revisions and contralateral ACL rupture [7].

More recent studies have focused on the impact of the difference between the lateral and medial PTS (i.e., the differential posterior tibial slope-dPTS), as measured from the lateral and medial posterior tibial condyles, respectively. In more detail, they have suggested that dPTS may be a stronger predictor of graft failure than absolute medial or lateral PTS values alone [1]. Although the association between dPTS and graft rupture is recognised, its potential influence on function and patient-reported outcomes (PROMs) following revision ACL reconstruction is unclear. Outcomes following revision ACL reconstruction surgery are generally inferior compared to primary ACL reconstruction, with, for example, the Knee injury and Osteoarthritis Outcome Score (KOOS) quality of life domain being as low as 48 ± 21 [8]. In this context, it could be important to identify anatomical factors that may contribute not only to graft failure but to poorer outcomes after revision surgery. A clearer understanding of the relationship between dPTS and postoperative PROMs may help identify patients at risk of suboptimal outcomes. This, in turn, could inform surgical decision-making including the use of adjunctive procedures aimed at optimising knee biomechanics and improving outcomes.

The purpose of this single-centre retrospective study was to investigate the impact of dPTS on PROMs following revision ACL reconstruction. Our hypothesis was that increased dPTS would be associated with inferior postoperative PROMs and that the proposed tibial slope assessment method yields reliable results. We therefore sought to (i) assess the relationship between differential PTS and PROMs at a minimum of one year following revision ACL surgery, considering both the absolute postoperative score and the change from preoperative baseline, and (ii) evaluate the intra- and interobserver reliability of differential PTS measurements. 

## 2. Methods

The prognostic cohort and reliability study was registered locally at our institution (CAE/QI 226), and Guidelines for Reporting Reliability and Agreement Studies (GRRAS) were followed [9]. We considered adult patients (over 18 years old) who underwent revision ACL reconstruction. A minimum of 1-year postoperative PROM data was required for a patient to qualify for inclusion, in addition to a 3 Tesla Magnetic Resonance examination (Philips Healthcare, Amsterdam, The Netherlands) with the appropriate sequences. We excluded patients who were diagnosed with knee osteoarthritis and/or ongoing meniscal or cartilage pathology, thus potentially affecting the postoperative clinical outcomes.

### 2.1. Primary Outcome Assessment

The primary outcome of the study was the change in mean Knee injury and Osteoarthritis Outcome Score (KOOS) from preoperative assessment to postoperative follow-up (minimum 1 year). The KOOS is a validated PROM that can be used to evaluate knee pathology and to monitor its course over time. The KOOS comprises 42 items across five independently scored subscales: Symptoms (7 items), Pain (9 items), Activities of Daily Living (17 items), Sport and Recreation Function (5 items), and knee-related Quality of Life (4 items). Each subscale is scored from 0 (extreme knee problems) to 100 (no knee problems).

For the interpretation of postoperative improvement, the following minimal important change (MIC) values were applied [10,11]: 7.3 points for Symptoms, 7.2 points for Pain, 7.9 points for Activities of Daily Living, 19.6 points for Sport and Recreation Function and 22.1 points for knee-related Quality of Life subscales.

### 2.2. Secondary Outcome Measures

**Figure 1:**
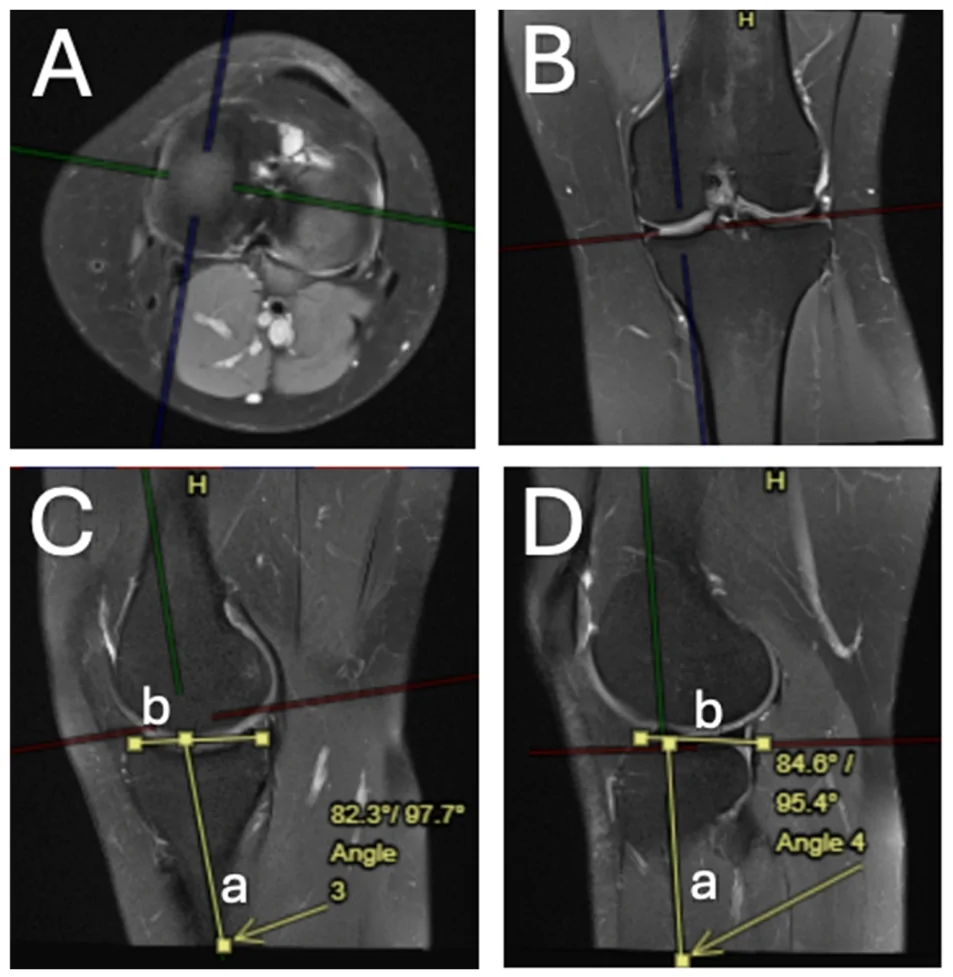
Proton density turbo spin echo (PD-TSE)-weighted magnetic resonance images of the knee demonstrating posterior tibial slope measurement. Image orientation was referenced to the midline of the medial tibial plateau using (**A**) axial and (**B**) coronal images. Posterior tibial slope was measured on sagittal images by drawing (a) a line along the anatomical axis of the tibia and (b) a line along the subchondral articular surface of the tibial plateau. Measurements are shown for (**C**) the medial tibial plateau and (**D**) the lateral tibial plateau.

**Figure 2:**
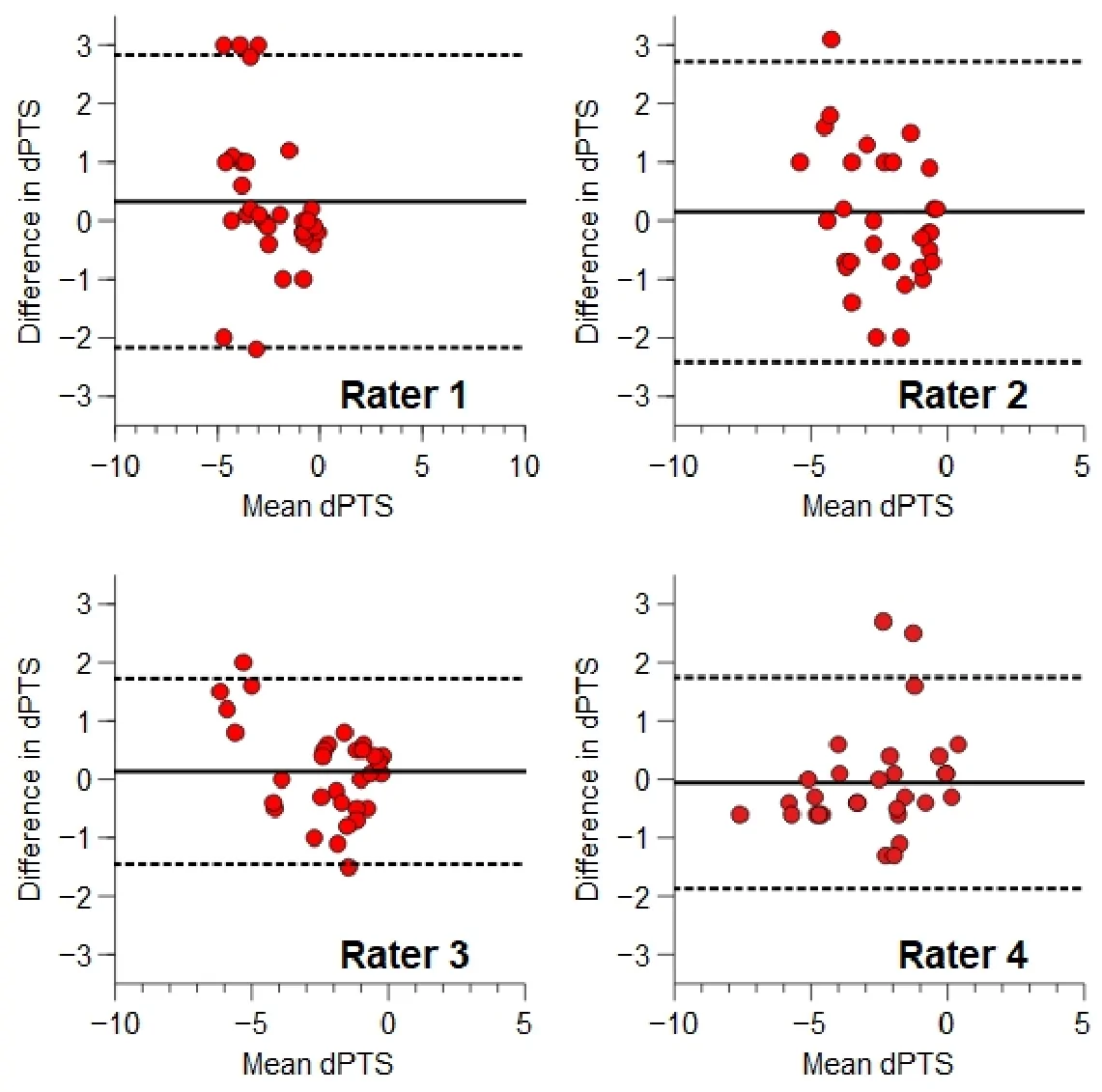
Bland–Altman plots demonstrating the extent of internal agreement between the measurements performed by the four raters.

The secondary outcomes of the study were (i) the intra- and interobserver variability of dPTS measurement using a previously published MRI-based method (Figure 1), (ii) the correlation between the changes in KOOS subscale scores and differential posterior tibial slope (dPTS), (iii) the correlation between postoperative KOOS and dPTS, and (iv) ACL graft re-ruptures. Four orthopaedic surgeons independently measured the dPTS from the patients’ preoperative MRI scans using a previously described method [12]. In addition, the raters were blinded to clinical outcomes and each other’s assessments. Each of the assessors evaluated the scans twice (two weeks apart) and the Bland–Altman plots were then generated for each of the raters to depict internal agreement (Figure 2).

Subsequently, Bland–Altman analysis was performed, including a linear regression of the differences against the averages, to assess whether the agreement between raters was dependent on the magnitude of the measurements (i.e., to detect proportional bias) and to evaluate the presence of any systematic bias (constant error). The regression line tests whether the slope differs significantly from zero (indicating proportional bias) and whether the intercept (mean difference) differs significantly from zero (indicating constant systematic bias). Of note, no significant constant bias was found (mean difference not significantly different from zero, *p* > 0.05). The average of the measurements was used for subsequent analyses of agreement and reliability. Bland–Altman analyses were also conducted for isolated medial slope and isolated lateral slope (see Supplementary Materials).

Subsequently, the interobserver variability was calculated by means of the intra-class correlation coefficient (ICC). Thresholds for the ICC are ICC < 0.50 to indicate poor interobserver reliability, 0.5 to 0.75 as being “moderate”, 0.75 to 0.90 as “good agreement” and 0.90+ as indicating excellent agreement [13]. The inter-rater reliability of the dPTS measure was evaluated using the ICC based on the two-way fixed effects model (absolute agreement), with patients as random effects and the four raters as fixed effects. The single-measures ICC (reliability of a single rater) was 0.46 (95% CI: 0.28–0.64), indicating moderate inter-rater reliability. The average-measures ICC (reliability of the mean rating from all four raters) was 0.77 (95% CI: 0.60–0.88), indicating good inter-rater reliability. Both estimates were statistically significant (*p* < 0.001). The single-measures ICC for isolated medial slope was 0.59 (95% CI: 0.42–0.74), indicating moderate inter-rater reliability. The average-measures ICC for isolated medial slope was 0.85 (95% CI: 0.74–0.92), indicating good inter-rater reliability. Similarly, the single-measures ICC for isolated lateral slope was 0.46 (95% CI: 0.28–0.64), indicating moderate inter-rater reliability. The average-measures ICC for isolated lateral slope was 0.77 (95% CI: 0.61–0.88), indicating good inter-rater reliability.

### 2.3. Statistics

The primary analysis was to assess the linear correlation between the mean dPTS and difference between pre- and postoperative KOOS subscale scores. This was assessed by means of the Pearson correlation coefficient. To aid interpretation [14], absolute correlation coefficients <0.3 indicate a weak relationship, 0.30 to 0.5 as moderate, and >0.5 as strong [15].

A pre-study power calculation indicated that a minimum of *n* = 29 patients was required to achieve 80% power to detect a moderate correlation of *r* = 0.5 between dPTS and the change in KOOS subscale scores (α = 0.05, two-sided). This sample size would also provide more than 80% power to detect a medium-sized change in the KOOS subscales (assumed Cohen’s *d* = 0.6). With *d* = 0.6, there is an approximately a 75% probability that a randomly selected patient will have a higher postoperative score than their own preoperative score (assuming normally distributed difference scores). Pearson’s correlation coefficient was used to assess the degree of correlation and the mean difference between pre- and postoperative scores was examined using a paired-samples *t*-test.

In addition to a change score analysis, an ANCOVA-style regression was performed to investigate the relationship between dPTS and postoperative KOOS subscale scores after controlling for KOOS baseline subscale as a covariate. Two-sided statistical tests were conducted and *p* < 0.05 was used to indicate statistical significance. All analyses were conducted using SPSS software version 31 (SPSS Inc., Chicago, IL, USA). Furthermore, the assumptions underlying the parametric analyses were evaluated. For the paired-samples *t*-tests, approximate normality of the paired difference scores was assessed by visual inspection of Q–Q plots. For the ANCOVA-style regression analyses, approximate normality of the residuals was assessed using the same approach. No material departures from normality were observed.

Not only did we assess the mean differential tibial slope in relation to the delta KOOS scores, but we also assessed the mean medial and mean lateral PTS and the difference between pre- and postoperative KOOS subscale scores as part of our secondary analyses. 

## 3. Results

**Figure 3:**
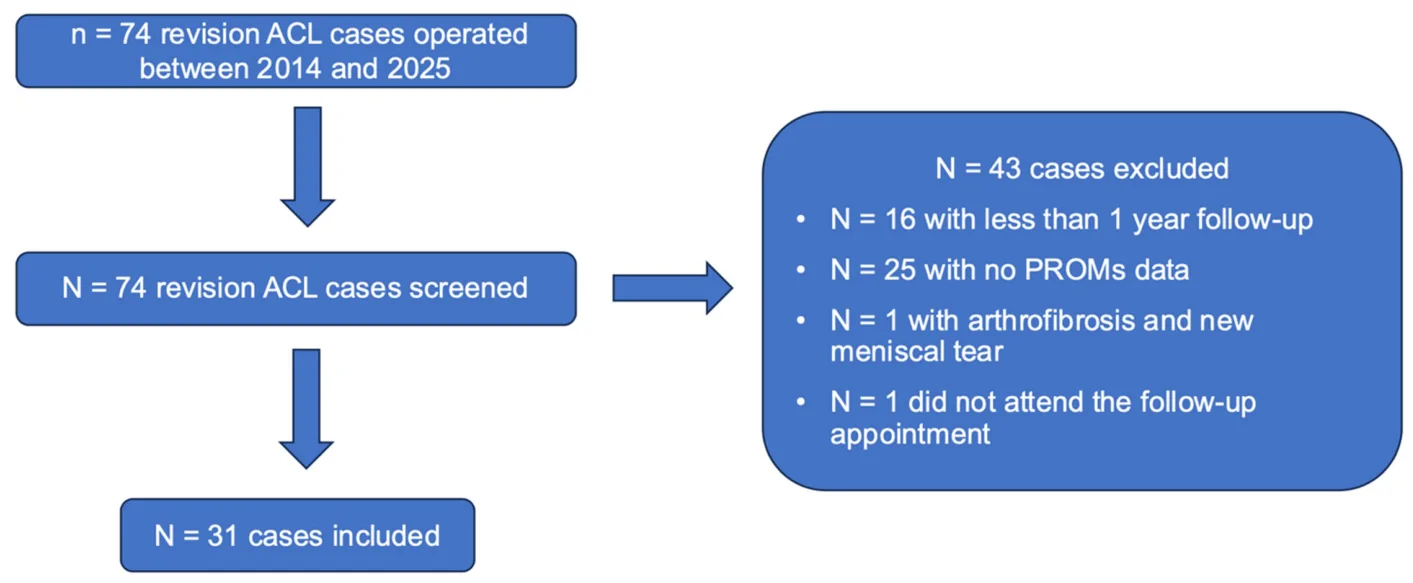
Flow chart of the study selection procedure. PROMs = patient-reported outcome measures.

Thirty-one patients who underwent revision ACL surgery between 2019 and 2024 were eligible for inclusion (Figure 3; Table 1 and Table 2). Further details on the revision procedures are presented in Table 2.

### 3.1. Primary Outcome Assessment

The average postoperative follow-up was at 1.68 years (range 1–5 years; 95% CI: 1.31 to 2.05). Postoperative KOOSs were significantly higher than preoperative scores, with the MIC being surpassed for all KOOS subscales except for the quality-of-life component (Table 3). 

### 3.2. Secondary Outcome Assessment

The mean average dPTS as −2.52 (SD = 1.43) (range −5.4 to −0.4). Mean differential PTS (dPTS) was significantly correlated with postoperative change in KOOS Symptoms (*r* = 0.472, 95% CI 0.141 to 0.708, *p* = 0.007), KOOS Pain (*r* = 0.612, 95% CI 0.329 to 0.794, *p* < 0.001), KOOS Sport and Recreation Function (*r* = 0.383, 95% CI 0.033 to 0.649, *p* = 0.034) and KOOS Quality of Life (*r* = 0.454, 95% CI 0.119 to 0.696, *p* = 0.010), but significance was not obtained for KOOS Daily Living (*r* = 0.330, 95% CI −0.028 to 0.613, *p* = 0.070). These relationships are shown in Figure 3. In contrast, the correlation between the dPTS for individual raters and change in KOOS subscales measurements showed notable variation (see Table 4) consistent with the lower single-measures ICC (single rater reliability). 

In an analysis of covariance regression analysis, mean differential posterior tibial slope (dPTS) was significantly related to postoperative KOOS Pain scores after controlling for preoperative KOOS Pain scores ($\hat{\beta }$ = 7.87, *t* = 4.931, *p* < 0.001), where $\hat{\beta }$ = denotes the estimated regression coefficient for the differential posterior tibial slope. This finding was replicated over Rater 1 (*p* = 0.004), Rater 2 (*p* = 0.001), Rater 3 (*p* < 0.001), and Rater 4 (*p* = 0.022). 

Similarly, mean differential PTS was significantly related to postoperative Activities of Daily Living after controlling for preoperative Activities of Daily Living scores ($\hat{\beta }$ = 3.86, *t* = 3.02, *p* = 0.005), and this finding was replicated over Rater 1 (*p* = 0.004), Rater 2 (*p* = 0.002), Rater 3 (*p* < 0.001), but not for Rater 4 (*p* = 0.809).

Mean differential PTS was significantly related to postoperative Sport and Recreation Function scores after controlling for preoperative scores ($\hat{\beta }$ = 6.87, *t* = 2.13, *p* = 0.042), and this finding was replicated over Rater 1 (*p* = 0.049), Rater 2 (*p* = 0.043), and Rater 3 (*p* = 0.011), but not for Rater 4 (*p* = 0.659). 

Mean differential PTS was significantly related to postoperative knee-related Quality of Life after controlling for preoperative scores ($\hat{\beta }$ = 8.00, *t* = 2.97, *p* = 0.006), and this finding was replicated over Rater 1 (*p* = 0.033), Rater 2 (*p* < 0.001), and Rater 3 (*p* = 0.002), but not for Rater 4 (*p* = 0.462). 

Mean differential PTS was significantly related to postoperative Other Symptoms scores after controlling for preoperative scores ($\hat{\beta }$ = 6.31, *t* = 3.69, *p* < 0.001), and this finding was replicated over Rater 1 (*p* = 0.004), Rater 2 (*p* = 0.002), and Rater 3 (*p* = 0.001), but not for Rater 4 (*p* = 0.278). 

Mean isolated medial slope was not significantly related to any changes in KOOS domains (Symptoms *r* = −0.276, *p* = 0.133; Pain *r* = −0.292, *p* = 0.111, Daily Living *r* = −0.025, *p* = 0.892, Sport and Recreation *r* = −0.192, *p* = 0.301, Quality of Life *r* = −0.041, *p* = 0.828). The same broad conclusions apply to the individual-level raters (see Supplementary Materials, Table S1). Similarly, mean isolated lateral slope was not significantly related to any changes in KOOS domains (Symptoms *r* = −0.022, *p* = 0.904; Pain *r* = 0.046, *p* = 0.805; Daily Living *r* = 0.151, *p* = 0.417; Sport and Recreation *r* = 0.033, *p* = 0.862; Quality of Life *r* = 0.241, *p* = 0.192). The corresponding 95% confidence intervals for the mean dPTS, mean medial PTS and mean lateral PTS are provided in Supplementary Table S2. The same broad conclusions apply to the individual-level raters (see Supplementary Materials, Table S3). Similarly, regression analyses controlling for preoperative KOOSs show that neither mean isolated medial slope nor mean isolated lateral slope were significantly related to the postoperative outcomes on any domain.

## 4. Discussion

The main finding of this study was that increased dPTS was associated with poorer PROMs (i.e., change in KOOS) following revision ACLR. A greater slope asymmetry (more extreme dPTS) demonstrated a moderate association with poorer improvements in KOOS Pain, Symptoms and Quality of Life subscale scores at a minimum of 1-year postoperative follow-up. Importantly, this relationship was observed when considering the change in PROMs, rather than the absolute postoperative scores, suggesting that the asymmetric bone morphology of increased dPTS may be associated not only with biomechanical risk factors for ACL graft failure, but also with poorer patient-reported functional outcomes.

Statistically significant postoperative improvements were observed across all KOOS domains, but the extent of clinically meaningful improvement was less uniform. Using the prespecified MIC values adopted in this study, the improvement in knee-related Quality of Life (21.4 points) was close to, but slightly below, the selected threshold of 22.1 points. This should, however, be interpreted with caution, as MIC estimates for KOOS are context-dependent and may vary according to the study population, follow-up setting, and estimation method.

Increased PTS has been shown in biomechanical cadaveric studies to cause greater anterior tibial translation and higher ACL graft strain [3,16]. Clinical evidence assessing the relationship between PTS and ACL reconstruction failure appears to support the above findings [17]. Recent analyses have suggested that dPTS, reflecting the asymmetry between the slope in medial and lateral compartments, may be a more relevant parameter [1]. The findings of the present study support this and suggesting a relationship between dPTS and PROMs following revision ACLR. Whilst previous studies of revision ACL reconstruction cohorts have found that PTS had no significant impact on subjective outcomes, including KOOS [18,19], these studies also only analysed medial or lateral PTS rather than dPTS. The findings of the present study also found no clear relationship between medial and/or lateral PTS and PROMs, and the cohort appears to be representative of revision ACL populations with postoperative KOOS subscale scores consistent with a large registry-based study [8], which showed similar improvements in KOOS subscales all exceeding the MCID. 

With respect to revision ACLR graft survivorship, previous large-scale studies have demonstrated relatively low re-rupture rates ranging from 3 to 15% over short-to-intermediate follow-up [20,21]. The absence of graft failures in the present small cohort with short-term follow-up is in keeping with the existing literature. Importantly, the findings presented in this study reinforce the concept that ACL graft integrity alone may be too simplistic a measure, highlighting the importance of PROMs. 

A key methodological challenge in measuring dPTS is accuracy, since standard radiographic measurements are prone to error due to tibial rotation [22]. Magnetic resonance imaging (MRI) provides a more accurate, true sagittal assessment of tibial slope [12], yet correlations between MRI-based measurements and long-term outcomes remain underexplored. Using MRI-based measurements, this study allowed accurate assessment of dPTS with reduced susceptibility to radiographic rotational error [23]. However, the reliability findings require a nuanced interpretation. Individual measurements demonstrated moderate single-rater interobserver reliability, whereas good reliability was observed when measurements were averaged across four raters. This distinction is clinically important because routine practice may more closely resemble the single-rater setting than the averaged-rater research setting. Accordingly, MRI-based assessment of dPTS may have value in preoperative evaluation, but the moderate single-rater reliability observed in this study should be considered when interpreting individual measurements. In selected cases, corrective osteotomy to normalise the tibial plateau geometry [24] may warrant consideration; however, further prospective studies are required to define appropriate thresholds and determine whether slope-correcting interventions improve patient-reported outcomes in the revision setting. 

A point of interest was the variability in the strength of the associations between dPTS and KOOS change scores across the individual raters. In Table 4, some raters showed weak or non-significant associations, whereas stronger and more consistent associations were observed for others, particularly Rater 3. This pattern is consistent with the moderate single-rater interobserver reliability observed for dPTS. The clearer associations observed when measurements were averaged across raters are likely explained by a reduction in random measurement error, leading to enhanced stability of the dPTS estimate under research conditions. While this supports the scientific value of averaging repeated measurements, it also suggests that the robustness and generalisability of the findings may be reduced in routine clinical settings where dPTS would more typically be measured by a single observer.

## 5. Study Limitations and Implications for Future Research

This study has several limitations. Its retrospective design and single-institution setting may limit the findings being generalised and may have introduced selection bias. 

MRI offers some advantages over conventional radiography in visualising tibial anatomy and helps improve measurement accuracy, but rotational malalignment cannot be completely excluded without computed tomography. In addition, the short-term follow-up period precluded evaluation of long-term outcomes such as degenerative change and early osteoarthritis development. The sample size was also modest, limiting the ability to assess the effect of dPTS on graft failure. We also wish to underline that patients underwent a wide variety of concomitant procedures (e.g., LEAT, meniscal surgery and high tibial osteotomies), and therefore the observed associations in the current paper should not necessarily be interpreted as representing an independent effect of differential posterior tibial slope given the limited sample size.

The ANCOVA-style regression analyses adjusted only for baseline KOOS values. Other clinically relevant variables, including age, sex, BMI, graft type, number of previous ACL procedures, meniscal status, cartilage pathology, osteotomy and concomitant LET, were not incorporated into the models. Given the modest sample size, a more comprehensive multivariable model was considered to be at risk of overfitting and unstable estimation. Consequently, residual confounding cannot be excluded and may have influenced the observed associations between dPTS and postoperative PROMs.

In addition, because this was an exploratory observational study rather than a confirmatory hypothesis-testing study, no formal adjustment for multiple comparisons was applied. Nevertheless, the number of analyses performed across KOOS domains may increase the risk of type I error inflation, and the findings should therefore be interpreted with caution. 

Furthermore, average dPTS measurements across the four assessors/raters were used for the primary analyses. While averaging may reduce random measurement error and improve estimate stability under research conditions, the individual-rater analyses demonstrated notable variability, consistent with the moderate single-rater interobserver reliability. This may limit the robustness and generalizability of the findings, where dPTS would be more typically measured by a single observer. Given the moderate single-rater reliability observed in this study, future work should explore whether the development and training in standardised training protocols can improve single-rater reliability, we emphasise that implementing a standardised training protocol for clinicians would be of the essence for a reliable evaluation in the clinical setting. Future developments in automated image-analysis and machine-learning-based measurement tools may also help improve accuracy, precision and reproducibility of dPTS assessment.

Additionally, it should be noted that the order of MRI evaluations was not randomised, and therefore a potential order effect (i.e., learning effect, fatigue effect) might have been introduced into the results of the study. 

As the cohort was derived from a specialist centre managing complex revision ACL cases, the findings may not be generalisable to centres with a different case-mix, surgical expertise, or use of adjunctive procedures.

Future research projects should additionally compare MRI-derived dPTS measurements with conventional standing radiographic measurements. Such future research includes retrospective validation studies, followed by prospective multi-centre studies to assess the relationships between measurement approaches and clinically relevant outcomes including PROMs. Furthermore, future papers should prioritise large, multicentre, prospective studies to validate the threshold value of the dPTS in predicting poor PROMs and explore the efficacy of corrective high tibial osteotomies designed to reduce the differential slope as a component of revision ACL reconstruction. In addition, large-scale studies with longer-term follow-up would help determine whether clinically useful dPTS thresholds can be developed and validated, and would allow assessment of the influence of high tibial osteotomies on PROMs.

## 6. Conclusions

The results of the current study suggest that greater differential posterior tibial slope is associated with poorer patient-reported outcomes. MRI-based assessment of dPTS demonstrated moderate single-rater and good average-rater interobserver reliability. These findings are encouraging and could potentially aid in preoperative risk stratification and patient management. However, before clinical recommendation can be recommended, there is a need for further prospective validation studies and a need to assess whether addressing excessive dPTS improves PROMs and graft survivorship.

## Figures and Tables

**Table 1:** Baseline characteristics of the included patients.

Characteristic	Mean Value (SD) (Range)
Age (years)	31.75 (6.67) (18 to 44)
Laterality (Right/left)	22/9
Height (cm)	175.9 (8.36) (161 to 193)
Weight (kg)	85.26 (16.78) (56 to 121)
BMI (units)	27.58 (5.41) (20.04 to 42.92)
Sex (males/females)	23/8

SD = Standard Deviation.

**Table 2:** Intervention characteristics related to the revision procedure of the included patients.

Participant ID	Graft Type	Supplemental Procedure
1	Ipsilateral HS autograft	MOWHTO and lateral meniscal repair
2	Ipsilateral BTB autograft	Lateral meniscal repair and LET
3	Ipsilateral BTB patella tendon graft	LET
4	Ipsilateral QT autograft	MCWHTO
5	Ipsilateral BTB autograft	Medial meniscal repair, chondroplasty and LET
6	Ipsilateral QT autograft	Lateral meniscal root repair, RAMP lesion repair and LET
7	Ipsilateral BTB	Lateral meniscal root repair, RAMP lesion repair and LET
8	Ipsilateral BTB autograft	Medial meniscal repair and LET
9	Ipsilateral BTB autograft	Medial meniscus root repair and LET
10	Ipsilateral HS autograft	Lateral meniscus repair
11	Contralateral QT autograft	Removal of metalwork from previous slope changing osteotomy and revision LET
12	Ipsilateral BTB autograft	Partial lateral meniscectomy and LET
13	Ipsilateral BTB autograft	Medial meniscal repair
14	Ipsilateral HS autograft	lateral root repair, LET, partial medial meniscectomy, intercondylar notchplasty
15	Ipsilateral BTB autograft	Medial meniscal debridement and LET
16	Ipsilateral QT autograft	MOWHTO, medial meniscal root repair and lateral meniscal root repair and LET
17	Ipsilateral QT autograft	Partial meniscectomy and MOWHTO
18	Contralateral HS autograft	Medial and lateral meniscal repairs, LET
19	Ipsilateral BTB autograft	Medial meniscal repair and lateral meniscal root repair
20	Ipsilateral BTB autograft	Partial medial meniscectomy and MOWHTO
21	Ipsilateral BTB autograft	Lateral meniscal repair and LET
22	Ipsilateral BTB allograft	LET
23	Ipsilateral BTB autograft	-
24	Ipsilateral BTB autograft	Medial partial meniscectomy and LET
25	Ipsilateral HS autograft	Medial meniscal repair, LET
26	Contralateral HS autograft	Medial and lateral meniscal repairs
27	Ipsilateral BTB autograft	Medial meniscus repair and LET
28	Ipsilateral BTB autograft	Lateral meniscal repair
29	Ipsilateral QT autograft	Lateral meniscal allograft transplant
30	Ipsilateral BTB autograft	Partial medial meniscectomy + chondroplasty medial femoral condyle + LET
31	Ipsilateral QT autograft	Anterior closing wedge slope reduction high tibial osteotomy

BTB = Bone-patellar tendon-Bone; QT = Quadriceps Tendon; HS = Hamstrings; LET = Lateral extra-articular tenodesis; MCWHTO = Medial Closing Wedge High Tibial Osteotomy; MOWHTO = Medial Opening Wedge High Tibial Osteotomy.

**Table 3:** Mean (SD) (95% Confidence Interval) KOOS subscale scores before and after revision ACL surgery and change.

KOOS Subscale	Preoperative	Postoperative	Mean Difference	*p* Value
Other Symptoms	62.1 (18.68) (55.26 to 68.96)	71.4 (15.682) (65.60 to 77.10)	9.2 (23.47) (0.63 to 17.85)	0.036
Pain	67.5 (17.78) (60.94 to 73.98)	80.1 (16.36) (74.08 to 86.09)	12.6 (22.43) (4.40 to 20.86)	0.004
Activities of Daily Living	79.5 (15.78) (73.68 to 85.26)	90.3 (11.39) (86.15 to 94.50)	10.9 (17.45) (4.46 to 17.26)	0.002
Sport and Recreation Function	38.4 (24.47) (29.41 to 47.36)	64.4 (27.08) (54.48 to 74.34)	26.0 (31.09) (14.62 to 37.43)	<0.001
Knee-related Quality of Life	24.80 (17.57) (18.36 to 31.24)	46.21 (24.73) (37.14 to 55.28)	21.41 (25.24) (95% CI 12.16 to 30.67)	<0.001

KOOS = Knee injury and Osteoarthritis Outcome Score.

**Table 4:** Pearson correlation coefficient and statistical significance between the differential posterior tibial slope and the change in KOOS subscales at >1 year postoperatively for each rater. Δ = change score; ADL = Activities of Daily Living.

KOOS Subscale	Rater 1	Rater 2	Rater 3	Rater 4
Δ Symptoms	0.319 (*p* = 0.080)	**0.369** **(*p* = 0.041)**	**0.682** **(*p* < 0.001)**	0.177 (*p* = 0.340)
Δ Pain	0.331 (*p* = 0.069)	**0.465** **(*p* = 0.008)**	**0.698** **(*p* < 0.001)**	**0.426** **(*p* = 0.017)**
Δ ADL	0.208 (*p* = 0.261)	0.300 (*p* = 0.101)	**0.533** **(*p* = 0.002)**	0.065 (*p* = 0.728)
Δ Sport and Rec	0.229 (*p* = 0.215)	0.327 (*p* = 0.072)	**0.404** **(*p* = 0.024)**	0.255 (*p* = 0.436)
Δ Quality of Life	0.313 (*p* = 0.087)	**0.616** **(*p* < 0.001)**	**0.468** **(*p* = 0.008)**	0.145 (*p* = 0.436)

## Data Availability

The data presented in this study are available on request from the corresponding author.

## Supplementary Materials

The following supporting information can be downloaded at: https://www.mdpi.com/article/10.3390/jcm15187125/s1, Figure S1: Medial Slope; Figure S2: Lateral Slope; Table S1: Pearson correlation coefficient and statistical significance between the Medial Slope and the change in KOOS subscales at >1 year post-operatively for each Rater. Δ = change score; ADL = Activities of Daily living; Table S2: Pearson correlation coefficients with 95% confidence intervals for associations between mean tibial slope measures and change in KOOS subscale scores; Table S3: Pearson correlation coefficient and statistical significance between the Lateral Slope and the change in KOOS subscales at >1 year post-operatively for each Rater. Δ = change score; ADL = Activities of Daily living.
