# Supplementary material for: The Impact of Differential Posterior Tibial Slope on Clinical Outcomes Following Revision ACL Surgery: A Retrospective Single-Centre Study
Source: J Clin Med. 2026 Sep 14;15(18):7125. doi: 10.3390/jcm15187125 (PMC13607150; doi:10.3390/jcm15187125)
Supplement: Supplementary file 1 [file jcm-15-07125-s001.zip › jcm-4351293-supplementary.pdf]

Figure S1. Medial Slope

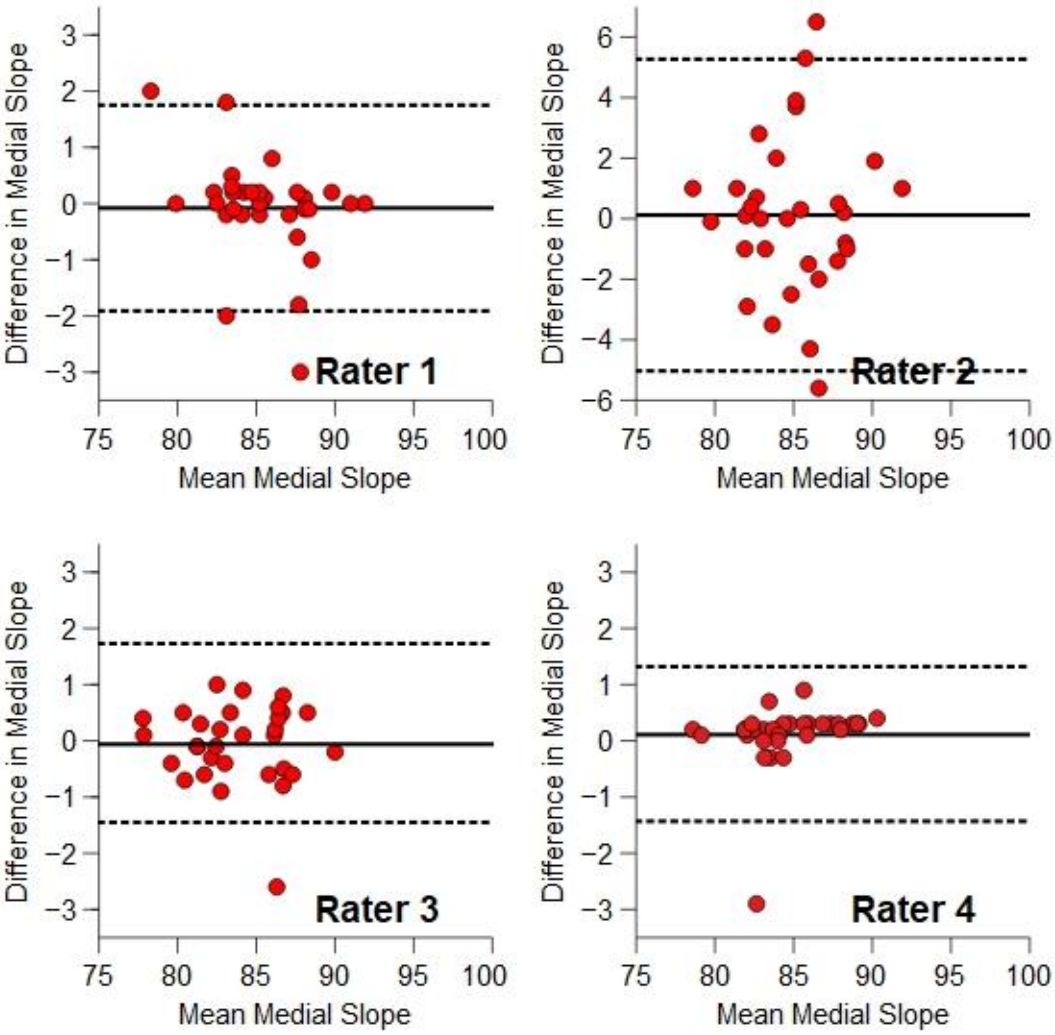

Table S1. Pearson correlation coefficient and statistical significance between the Medial Slope and the change in KOOS subscales at >1 year post-operatively for each Rater. Δ = change score; ADL = Activities of Daily living

KOOS subscale	Rater 1	Rater 2	Rater 3	Rater 4
Δ Symptoms	-0.259<br>(p = .160)	-0.188<br>(p = .312)	0.306<br>(p = .094)	-0.171<br>(p = .356)
Δ Pain	-0.200<br>(p = .280)	-0.153<br>(p = .412)	-0.500<br>(p = .004)	-0.119<br>(p = .523)
Δ ADL	-0.007<br>(p = .972)	0.088<br>(p = .637)	-0.240<br>(p = .194)	0.084<br>(p = .655)
Δ Sport and Rec	-0.108<br>(p = .563)	-0.109<br>(p = .558)	-0.393<br>(p = .029)	-0.024<br>(p = .900)
Δ Quality of Life	0.017<br>(p = .928)	-0.052<br>(p = .780)	-0.199<br>(p = .284)	0.107<br>(p = .566)

Figure S2. Lateral Slope

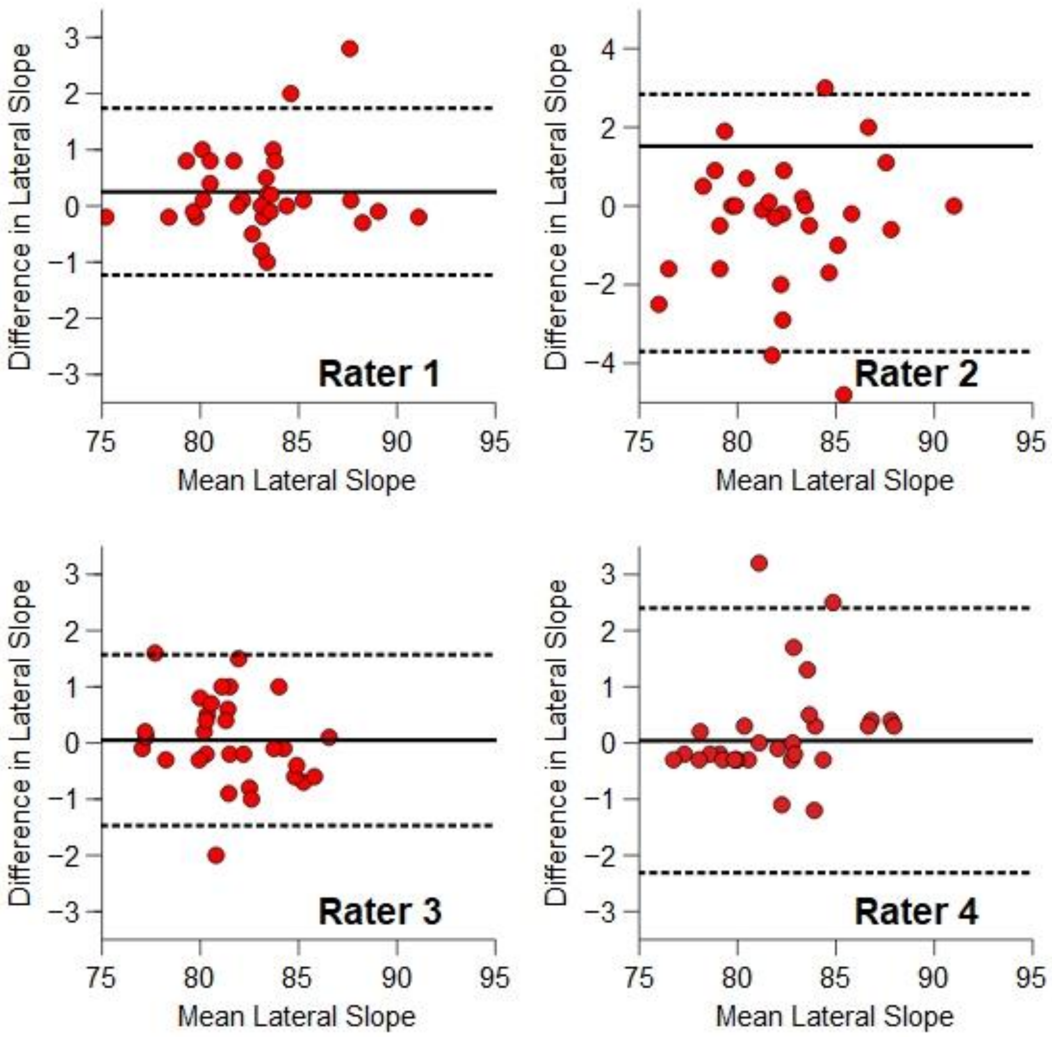

Table S2. Pearson correlation coefficients with 95% confidence intervals for associations between mean tibial slope measures and change in KOOS subscale scores.

Slope measure	KOOS change subscale	r	95% CI	p value
Mean dPTS	Δ Symptoms	0.472	0.141 to 0.708	.007
Mean dPTS	Δ Pain	0.612	0.329 to 0.794	<.001
Mean dPTS	Δ ADL	0.330	−0.028 to 0.613	.070
Mean dPTS	Δ Sport and Recreation	0.383	0.033 to 0.649	.034
Mean dPTS	Δ Quality of Life	0.454	0.119 to 0.696	.010
Mean medial PTS	Δ Symptoms	−0.276	−0.574 to 0.087	.133
Mean medial PTS	Δ Pain	−0.292	−0.586 to 0.070	.111
Mean medial PTS	Δ ADL	−0.025	−0.376 to 0.332	.892
Mean medial PTS	Δ Sport and Recreation	−0.192	−0.512 to 0.174	.301
Mean medial PTS	Δ Quality of Life	−0.041	−0.390 to 0.318	.828
Mean lateral PTS	Δ Symptoms	−0.022	−0.373 to 0.335	.904
Mean lateral PTS	Δ Pain	0.046	−0.313 to 0.394	.805
Mean lateral PTS	Δ ADL	0.151	−0.215 to 0.480	.417
Mean lateral PTS	Δ Sport and Recreation	0.033	−0.325 to 0.383	.862
Mean lateral PTS	Δ Quality of Life	0.241	−0.124 to 0.549	.192

CI = confidence interval; dPTS = differential posterior tibial slope; PTS = posterior tibial slope; KOOS = Knee injury and Osteoarthritis Outcome Score; ADL = Activities of Daily Living. Correlations are based on n = 31.

Table S3. Pearson correlation coefficient and statistical significance between the Lateral Slope and the change in KOOS subscales at >1 year post-operatively for each Rater. Δ = change score; ADL = Activities of Daily living

KOOS subscale	Rater 1	Rater 2	Rater 3	Rater 4
Δ Symptoms	-0.091<br>(p = .627)	-0.029<br>(p = .877)	0.126<br>(p = .499)	-0.045<br>(p = .810)
Δ Pain	-0.032<br>(p = .865)	0.065<br>(p = .727)	-0.096<br>(p = .609)	0.190<br>(p = .306)
Δ ADL	0.090<br>(p = .632)	0.202<br>(p = .276)	0.100<br>(p = .593)	0.070<br>(p = .710)
Δ Sport and Rec	0.006<br>(p = .974)	0.080<br>(p = .669)	-0.181<br>(p = .330)	0.161<br>(p = .388)
Δ Quality of Life	0.159<br>(p = .392)	0.240<br>(p = .193)	0.097<br>(p = .604)	0.237<br>(p = .199)
